# Pathways of IFN-alpha Activation in Patients with Cervical Intraepithelial Neoplasia (CIN)

**DOI:** 10.1055/s-0041-1735301

**Published:** 2021-10-20

**Authors:** Nelson Ranieri Tirone, Carolina Guissoni Campos, Kézia Jesus Aguiar Ferreira, Léticia Montes Stark, Jéssica Ferreira Vieira, Eddie Fernando Cândido Murta, Márcia Antoniazi Michelin

**Affiliations:** 1 Instituto de Pesquisa em Oncologia, Universidade Federal do Triângulo Mineiro, Uberaba, MG, Brazil; 2 Department of Gynecology and Obstetrics, Universidade Federal do Triângulo Mineiro, Uberaba, MG, Brazil; 3 Discipline of Immunology, Universidade Federal do Triângulo Mineiro, Uberaba, MG, Brazil

**Keywords:** interferon receptors, cervical intraepithelial neoplasia, interferon alpha, receptores de interferon, neoplasia intraepitelial cervical, interferon alfa

## Abstract

**Objective** The aim of the present study was to compare the local and systemic expression of the factors linked to the interferon alpha (IFN-α) activation pathway in different degrees of cervical intraepithelial neoplasia (CIN) and cervical cancer.

**Methods** A total of 128 patients with CIN I, CIN II, CIN III and cervical cancer was evaluated. The real-time polymerase chain reaction (RT-PCR) technique was used to evaluate the gene expression of *IFNR1* , *IFNR2* , IFN-α, oligoadenylate synthase (2'5′OAS), cytokine signal suppressor 1 ( *SOCS* ) 1, *SOCS3* , signal transducer and transcription activator 1 ( *STAT1* ), and *IRF9* from 128 biopsies. A total of 46 out of 128 samples were evaluated by flow cytometry for *IFNAR1* , *IFNAR2* , *STAT1* , *IRF7* and IFN-α in peripheral blood cells.

**Results** Patients with CIN II and III (63 samples) had a low local expression of *IFNR1* , but not *IFNR2* . Patients with some degree of injury showed high expression of *SOCS1* and *SOCS3* . Systemically, patients with CIN II and III (20 samples) had a significant increase in *IFNR1* , *IFNR2* , *STAT1* , *IRF7* , and IFN-α in helper, cytotoxic T lymphocytes, and in monocytes.

**Conclusion** Patients with high-grade lesions have increased systemic expression of IFN-α and its activation pathways in helper and cytotoxic T lymphocytes, as well as in monocytes due to an exacerbation of the immune response in these patients. This phenomenon is not accompanied by resolution of the lesion due to a defect in the IFN-α activation pathway that revealed by low local IFNAR1 expression and high local expression of *SOCS1* and *SOCS3* .

## Resumo

**Objetivo** O objetivo do presente estudo foi comparar a expressão local e sistêmica dos fatores ligados à via de ativação do interferon alfa (IFN-α) em diferentes graus de neoplasia intraepitelial cervical (NIC) e câncer cervical (CA)

**Métodos** Foram avaliados 128 pacientes com NIC I, NIC II, NIC III e CA. A técnica de reação de cadeia de polimerase em tempo real (RT-PCR, na sigla em inglês) foi realizada para avaliar a expressão gênica do receptor de interferon (IFNR) 1, IFNR2, IFN-α, 2′-5′-oligoadenilato sintetase (2'5′OAS), supressor de sinalização de citocina (SOCS)1, SOCS3, transdutor de sinal e ativador de transcrição 1 (STAT1) e fator regulador de interferon 9 (IRF9) das 128 biópsias. Das 128 amostras, 46 foram avaliadas por citometria de fluxo para IFNAR1, IFNAR2, STAT1, IRF7 e IFN-α em células de sangue periférico.

**Resultados** Pacientes com NIC II e III (63 amostras) tiveram baixa expressão local de IFNR1 mas não de IFNR2. Pacientes com algum grau de lesão apresentaram alta expressão de SOCS1 e SOCS3. Sistemicamente, os pacientes com NIC II e III (20 amostras) tiveram um aumento significativo de IFNR1, IFNR2, STAT1, IRF7 e IFN-α em linfócitos T auxiliares, citotóxicos e monócitos.

**Conclusão** Pacientes com lesões de alto grau apresentam expressão sistêmica aumentada de IFN-α e suas vias de ativação em linfócitos T auxiliares e citotóxicos, bem como em monócitos, devido à exacerbação da resposta imune nesses pacientes. Este fenômeno não é acompanhado pela resolução da lesão devido a um defeito na via de ativação do IFN-α que é revelado pela baixa expressão local de IFNR1 e alta expressão local de SOCS1 e SOCS3.

## Introduction

The possibility of early screening and treating neoplastic lesions was of considerable importance in reducing the incidence and deaths from invasive cervical cancer. Cervical intraepithelial neoplasms (CIN), which are preinvasive lesions, have different degrees. Grade I should be treated to preserve the cervix, while for grades II and III, local excision of the cervix has been considered as a therapeutic intervention because the latter has the potential for progression to invasive cancer (CA). 1 However, this imposes risks during pregnancy, such as increased prematurity and abortions. 2

Thus, alternatives for treatment have been evaluated, such as the use of interferons (IFNs), which have been recognized as extremely important cytokines for human health, since they have therapeutic potential against viral actions. 3 4 As a fundamental part of the immune response, interferon alpha (IFN-α) is able to inhibit exacerbated cell proliferation through its signaling pathways. It is also able to suppress the expression of oncogenes and to promote apoptosis. 5

A Janus kinase/tyrosine kinase (JAK/TYK) signaling cascade is activated when IFN-α binds to specific interferon receptors (IFNR1 and IFNR2). This results in the generation of interferon-stimulated genes (ISGs). Viral clearance begins with peripheral immune cells alerted by the cell in an antiviral state, along with transcription factors. 3 6

Transcription factors can act in the regulation of the cell cycle, apoptosis, and body defense. They are also present in homeostasis and immune cell development and are related to oncogenesis. As an example of transcription factors are the signal transducer and transcription activator (STAT) and interferon regulatory factor (IRF) family. 7 8 In addition, there are the regulators of the signaling pathways in the cellular machinery orchestrated by IFN-α. The cytokine signal suppressor (SOCS) is composed of SOCS1-SOCS7. Involved in the negative regulation of JAK/STAT signaling, the SOCS suppresses endogenous and prognostic signaling of IFN-α by viral infection. 9 10

Thus, the aim of the present study was to compare the expression of factors directly linked to the IFN-α activation pathway in different degrees of CIN and cervical cancer, locally and systemically, by flow cytometry and molecular analysis.

## Methods

### Research Subjects

The present study included women between 18 and 50 years old with CIN I ( *n* = 45), CIN II ( *n* = 30), CIN III ( *n* = 33), and cervical cancer ( *n* = 20). The inclusion criteria were absence of bleeding during the exam, no use of oral antibiotics, fungicides, or vaginal creams in the last 30 days; no sexual activity for at least 2 days before the collection day; and with no previous history of treatment for human papillomavirus (HPV). Patients with negative pathological results for CIN and HPV were included in the controls. All procedures performed followed the criteria proposed by the Research Ethics Committee (CEP, in the Portuguese acronym) under the protocol number 683-2006, with all patients having signed free and informed consent form.

For local analysis, biopsies were collected and stored in TRIzol (Invitrogen, Waltham, MA, USA) for subsequent RNA extraction and analysis via real-time polymerase chain reaction (RT-PCR) for the analysis of IFNR1, IFNR2, oligoadenylate synthase (2'5′OAS), IFN-α, STAT1, IRF9, SOCS1, and SOCS3. For the systemic analysis, peripheral blood was collected for verification by flow cytometry of IFNR1, IFNR2, IFN-α, STAT1, and IRF7.

### Flow Cytometry

Peripheral blood (PB) samples from the patients were taken and the cells were evaluated by flow cytometry (BD FACS Calibur TM cytometer, BD Biosciences, San Diego, CA, USA). The cytometry protocols were implemented according to those suggested by the manufacturer. Peripheral blood cells were evaluated for the following markers: CD3+CD4+ (T helper lymphocytes), CD3+CD8+ (T CTL lymphocytes), and CD14+ (monocytes).

Leukocytes were isolated from PB samples by centrifugation at 4°C, following the instructions of the manufacturer (FACS Lysing Solution, BD Biosciences, Franklin Lakes, NJ, USA). The cells were resuspended in phosphate-saline solution (PBS) for extracellular marking with anti-CD3, anti-CD4, anti-CD8 or anti-CD14. For intracellular labeling, the cells were incubated with the following antibodies labeled with IFNR1, IFNR2, IFN-α, STAT1 or IRF7.

In all experiments and in all patients, we used intracellular and extracellular negative control isotopes using fluorochromic conjugated antibodies according to the reference antibody. After intracellular staining, the cells were incubated at 4°C for 30 minutes and resuspended in 500μL of PBS for cytometric analysis in the cytometer. For the specific determination of cells corresponding to lymphocytes and macrophages, we identified the region to be analyzed by the construction of gates according to controls of relative size (direct dispersion; FSC) and granularity and complexity (lateral dispersion; SSC) in each experiment and for each patient.

### RT-PCR – Reverse Transcription and Polymerase Chain Reaction

The RNA was extracted from cervical biopsies using TRIzol reagent. cDNA synthesis was performed with Superscript III rt (Invitrogen, Waltham, MA, USA). Conventional PCR was performed with the PCR mixing solution (Invitrogen, Waltham, MA, USA). The samples were taken to a thermal cycler with the following programming: initial denaturation at 94°C for 3 minutes; amplification of 40 cycles at 94°C for 1 minute (denaturation), annealing temperature for 1 minute (hybridization) and at 72°C for 1 minute (polymerization); final polymerization: 72°C for 10 minutes. At the end of the amplification cycles, the reaction was stopped by cooling to 4°C. Primer sequences used were: Beta-actin, *(F) 5′-GTG GGG CGC CCC AGG CAC CA-3′, (R) 5′-CTC CTT AAT GTC ACG CAC GAT TTC-3′* , 56° C, 295 bp; IFNR1, ( *F) 5′-CTT TCA AGT TCA GTG GCT CCT CGC-3′, (R) 5′-TCA CAG GCG TGT TTC CAG ACT G-3′* , 57° C, 500 bp; IFNR2, *(F) 5′-GAA GGT GGT TAA GAA CTG GC-3′, (R) 5′-CCC GCT GT TCC TTC TAG GAC GG-3′* , 57° C, 105 bp; IFN-alpha, (F) *5 ´-ACTTTGGATTTCCCCAGGA-3 ´, (R) 5 ´-CAGGCACAGGGCTGTATT-3* , 59° C, 316 bp; SOCS1, *(F) 5′-ACG AGC ATC CGC GTG CAC TT3′, (R) 5′-AAG AGG CAG TCG AAG CTC TC-3* ', 64° C, 90 bp; SOCS3, *(F) 5′-GAA GAT CCT GGT GTT GA-3′, (R) 5′-TTC CGA CAG AGA TGC TGA AGA GT-3′* , 63° C, 70 bp; STAT1, *(F) 5′-AGG AAG CAC CAG AGC CAA TGG AAC-3′, (R) 5′-GAG CCC ACT ATC CGA GAC ACC TCG-3′* , 59° C, 150pb; IRF9, *(F) 5′-AGA GGA TGC CAT GCA GAA CTG-3′, (R) 5′-GCT CCC AAT GTC TGA ATG GAC-3′* , 63° C, 110 bp; 2'5′OAS, *(F) 5′-ACC TGG TTG TCT TCC TCA GTC C-3′, (R) 5′-GAG CCT GGT CCT CAA ACT TCA C-3′* , 54° C, 200 bp.

To visualize the products amplified by the RT-PCR reactions, the samples were submitted to 10% polyacrylamide gel electrophorese. Then, the gels were stained with silver nitrate. A positive standard control of 50 bp DNA Invitrogen ladder was used.

### Statistical Analysis

An electronic database was developed for statistical analysis. The variables were analyzed using GraphPad Prism 8.4 (GraphPad Software, San Diego, CA, USA). The values were subjected to the chi-squared statistical test for PCR and to the Kruskal-Wallis test with the Dunn post-test for cytometry values. Differences with *p* ≤ 0.05 were considered statistically significant.

## Results

**Fig. 1.**
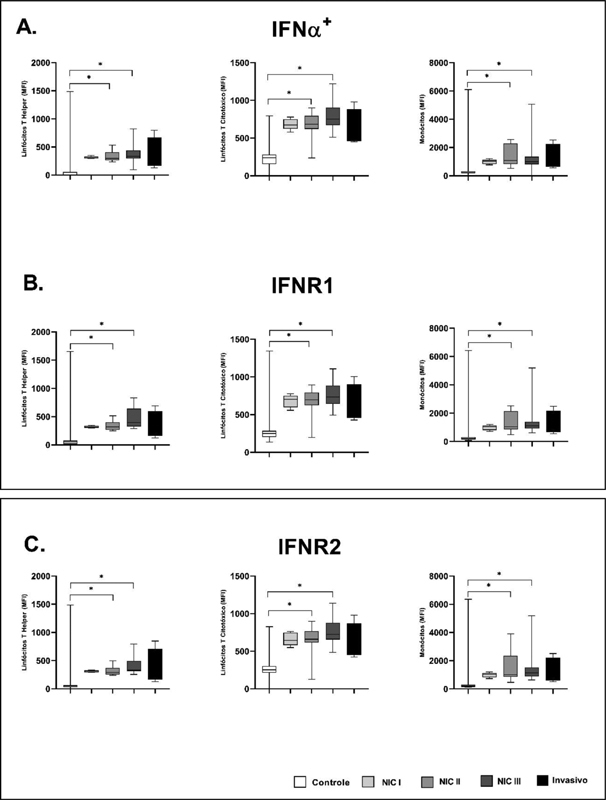
Mean intensity of flowering (MFI) in the CD14 + , CD8 + , and CD4+ populations of IFN-α, IFNR-1 and IFNR-2. A. IFN-α MFI in Control, NICII, NICIII and Invasive groups in the three cell populations. B. IFNR1 receptor MFI in the Control NICII, NICIII and Invasive groups in the three cell populations. C. IFNR2 receptor MFI in the Control NICII, NICIII and Invasive groups in the three cell populations. The results were achieved using the Kruskal-Wallis test with the Dunn post-test in GraphPad Prism 8.4. For the comparisons Control x CIN II and Control x CIN III in the graphs, significance was observed, with *p* < 0.05 (5%). Values represented in median (min-max).

A systemic analysis of the receptors and transcription factors expressed in different cell populations was carried out to better understand the cell activation process of the studied groups. The mean fluorescence intensity (MIF) was observed for T CTL lymphocytes (CD8 + ), T helper lymphocyte (CD4 + ), and monocytes (CD14 + ) ( Figure 1 ).

The MFI for IFN-α was higher in monocytes. When comparing the different study groups by cell type, a significant increase in CIN II and CIN III was observed in relation to Control in the populations of CD4+ ( *p* =0.0005), CD8+ ( *p* <0.0001), and mococyte cells/CD14+ ( *p* =0.0029) ( Figure 1A ).

For the IFNR1 and IFNR2 receptors ( Figure 1B, C ), in each cell population compared (CD4+, CD8+, and CD14+), the IFNR1 and IFNR2 receptors were increased in the CIN II and CIN III groups compared to the Control group IFNR1+ CD4+ ( *p* = 0.0002); IFNR1+ CD8+ ( *p* = 0.0011); IFNR1+ CD14+ ( *p* = 0.0011); IFNR2+ CD4+ ( *p* = 0.0003); IFNR2 ^+^ CD8 ^+^ ( *p* = < 0.0001); and IFNR2 ^+^ CD14+ ( *p* = 0.0009).

**Fig. 2.**
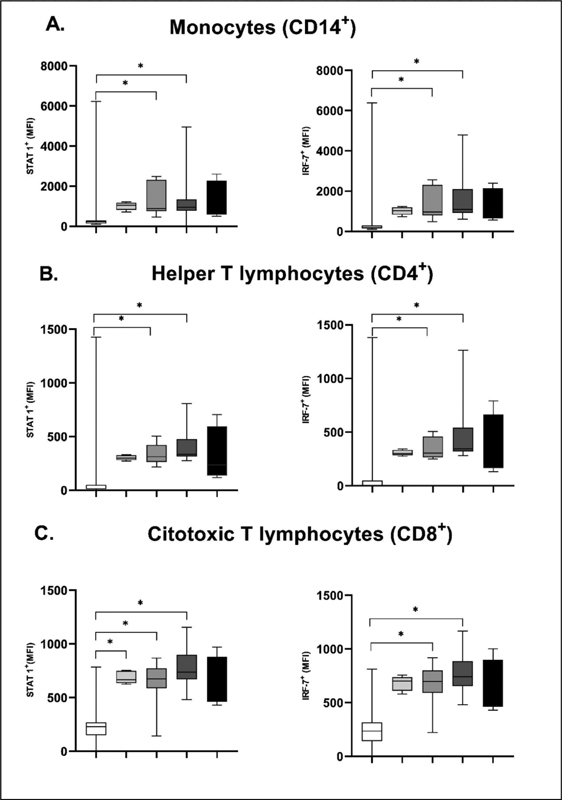
Mean intensity of flowering (MFI) in the CD14 + , CD8 + , and CD4+ populations of STAT1+ and IRF-7 + . A. IFN-α MFI in Control, NICII, NICIII and Invasive groups in the three cell populations. B. IFNR1 receptor MFI in the Control NICII, NICIII and Invasive groups in the three cell populations. C. IFNR2 receptor MFI in the Control NICII, NICIII and Invasive groups in the three cell populations. The results were analyzed using the Kruskal-Wallis test with the Dunn post-test in GraphPad Prism 8.4. For the comparisons Control x CIN II and Control x CIN III in the graphs, significance was observed, with *p* < 0.05 (5%). Values represented in median (min-max).

Transcription factors were also analyzed in the T helper lymphocytes, T CTL lymphocytes, and monocytes ( Figure 2 ). we observed a significant increase in CIN II and CIN III compared to the Control group ( Figure 2A ) for both STAT1 ( *p* = 0.0031) and IRF-7 ( *p* = 0.0008). The T helper and T CTL lymphocytes had a lower MFI than the monocyte population. We observed a significant increase in IRF7+ in the CIN II and CIN III groups compared to the Control group in the population of CD4+ and CD8+ cells in the IRF-7; IRF-7 + CD4+ ( *p* = 0.0002); IRF-7 + CD8+ ( *p* < 0.0001). Regarding STAT1, we observed a significant increase in CIN I, CIN II, and CIN III compared to Control for the two cell populations evaluated (CD4+ and CD8+) ( *p* = 0.0350) ( Figure 2B, C ) in the CD8 + , STAT1+CD4+ ( *p* = 0.0003); and STAT1 + CD8+ ( *p* < 0.0001) populations.

**Fig. 3.**
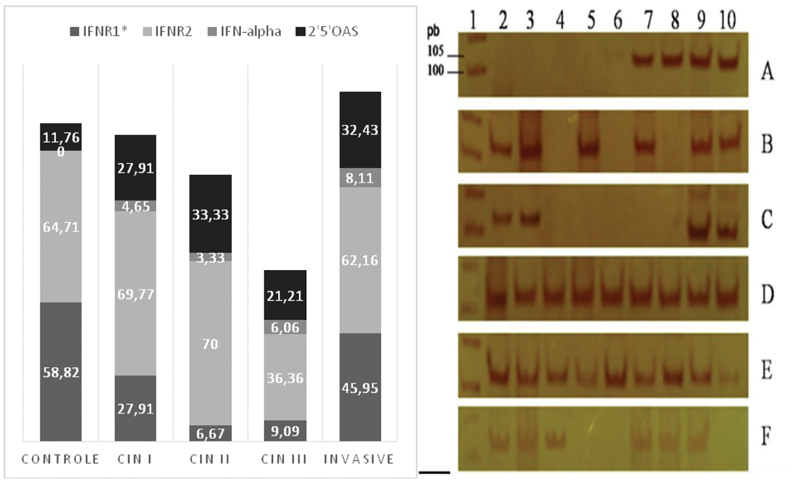
Graph representing the percentage of expression of the *IFNR1* , *IFNR2* , *IFN-alpha* , and *2'5′OAS* genes. A. Results of positivity of conventional PCR performed using the chi-squared statistical test. * *p* < 0.0001. B. Photograph of 10% polyacrylamide electrophoresis gel stained with silver nitrate for analysis of RT-PCR products for IFNAR-2. Samples A and B of CIN I; C CIN II samples; D, E, F CIN III and invasive samples.

Analyzing the local expression of IFN-alpha receptors and transcription factors, it was found that the IFNR2 receptor was expressed in approximately 70% of patients in the control groups, CIN I, CIN II and Invasive Cancer. Statistically significant, the IFNR1 receptor was expressed in approximately 10% of patients in the CIN II and CIN III groups. The control group showed no expression for IFN-alpha and the expression for the enzyme 2'5′OAS showed no difference between the groups ( Figure 3A ). The expression of the IFNR2 receptor can be observed with the presence of a fragment of molecular weight 105bp for positive patients ( Figure 3B ).

**Fig. 4.**
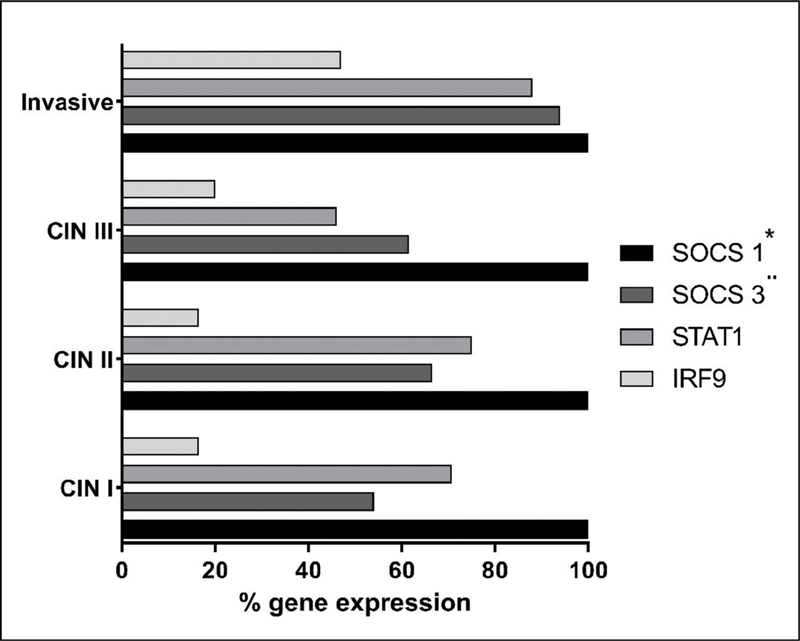
Graph representing the percentage of patients expressing SOCS1, SOCS3, STAT1 and IRF9. Positive results from conventional PCR performed using the chi-squared statistical test. * *p* <0.0001; ¨ *p* = 0.0085.

Transcription factors linked to IFN-α cell activation were observed for sites in each group. It was observed that SOCS1 was expressed in 100% of the samples evaluated from the four study groups (CIN I, CIN II, CIN III, and Inavise). SOCS 3 was expressed in 95% of the samples evaluated in the Invasive group; approximately 60% in the CIN III and CIN II groups, and approximately 55% in the CIN I. STAT1 was expressed in approximately 90% of the samples in the Invasive group, ~50% in CIN III, ~80% in CIN II, and ~70% in CIN I. And IRF9 was expressed in ~45% of the samples from the Invasive group and ~20% in the other grypos (CIN III, CIN II, and CIN I) had a higher percentage of patients who expressed all transcription factors ( Figure 4 ).

## Discussion

According to the analyzes performed, IFNR1 and IFNR2 were expressed in different ways in the cell subtypes analyzed in PB by flow cytometry. Literature data show that the concentration of the receptor on the cell surface and that the organization of the cell in space are important parameters that can cause different responses to IFN-α in individuals. 11

RT-PCR analyzes demonstrate a decrease in the expression of IFNR1 and IFNR2 in lesions, as the lesion progresses from CIN I to CIN III, with the expressions being reduced in relation to the non-existence of the lesion (Control group). However, its expression is increased in Invasive. In a previous study carried out in our group, it was observed that IFRNR1 and IFNR2 (mRNA) were poorly expressed in neoplastic biopsies when compared with control biopsies. 12

According to the guidelines that regulate cervical biopsies, such as the International Federation of Gynecology and Obstetrics, women with altered cytological and colposcopic results with suspicion of high-grade HPV or justifiable disease, are submitted to biopsies. 13 Thus, a disease-free control group could not be included in our study because women without colposcopic changes are not submitted to biopsy, an important condition for inclusion in the studied groups.

The density of receptors on the cell surface and the binding affinity of the IFN-α subtypes with them are directly related to the different responses triggered by the cytokine binding with the receptor. 14 Therefore, it may be that the number of women who expressed the receptors in the group of CINIII lesions and invasive carcinoma was not enough to generate a local IFN-α response.

The present study demonstrated that during the evolution of the neoplastic lesion, no alterations in the expression of IFN-α were observed. The same can be observed in a study that showed no gene expression of IFN-α in Control samples. 12 However, data from the literature demonstrate that IFN may be increased in viral infections 15 16 and demonstrate the various ways in which HPV oncoproteins interfere with the IFN-alpha activation system, interacting with signaling and signal transduction pathways and inhibiting the activation of transcriptional factors. Recently, the function of the E5 protein, which can suppress IFN signaling, was discovered,. 17

Our data also show that the CINI group has a high expression of STAT1 and SOCS1 and a low expression of IFNR1. The SOCS1 can exert an antitumor function by regulating chronic inflammation and mediating the phosphorylation of the tumor suppressor protein p53. 18

Our data also showed that, with the progression of the lesion, a decrease in the expression of SOCS3 is observed. In another study, a similar situation was demonstrated; however, regarding HPV positivity: a reduction in the expression of SOCS and SOCS3 in HPV positive tumors, whereas, in HPV negative tumors, the expression of SOCS was similar to that of the healthy control group. This negative regulation of SOCS can be mediated by HPV. 19

It was also possible to observe that an MFI by STAT1 flow cytometry was higher in the population of TCL than in the other studied populations. In an assay performed with HeLa cells, in which endocytosis of the IFNAR receptor was blocked, an insufficient presence of STAT1 was observed in the nucleus, and even after IFN-α stimulation the transcriptional performance dependent on this cytokine was inhibited. Therefore, blocking the endocytosis of IFNAR is likely to prevent the transcription of activated genes in advance, among which are essential genes to initiate antiviral and antiproliferative functions. 20

The presence of HPV alone in the uterine cervix can be an adjuvant in the negative regulation of IFNR1, being related to the different systemic and local expressions. Some factors, such as smoking, immune response, sexual behavior, multiple HPV infections, and viral load, are related to the progression from low-grade lesions to high-grade lesions. It is known that HPV can induce cell polarization for the anti-inflammatory profile through the production of cytokines with an immunoregulatory profile, thus favoring the progression of lesions. 13 21

## Conclusion

It is concluded that patients with high-grade lesions have a greater systemic expression of IFN-α and its activation pathways in helper and cytotoxic T lymphocytes, as well as in monocytes, due to an exacerbation of the immune response in these patients. This phenomenon is not accompanied by the resolution of the lesion due to a defect in the IFN-α activation path revealed by the low local expression IFNR1 and high local expression of SOCS1 and SOCS3.

## References

[1] Landy R Pesola F Castañón A Sasieni P Impact of cervical screening on cervical cancer mortality: estimation using stage-specific results from a nested case-control study Br J Cancer 2016 115 09 1140 1146 10.1038/bjc.2016.290 27632376 PMC5117785

[2] Kyrgiou M Athanasiou A Kalliala I EJ Paraskevaidi M Mitra A Martin-Hirsch P P Obstetric outcomes after conservative treatment for cervical intraepithelial lesions and early invasive disease Cochrane Database Syst Rev 2017 11 11 CD012847 10.1002/14651858.CD012847 29095502 PMC6486192

[3] Stark G R Kerr I M Williams B R Silverman R H Schreiber R D How cells respond to interferons Annu Rev Biochem 1998 67 227 264 10.1146/annurev.biochem.67.1.227 9759489

[4] Hervas-Stubbs S Perez-Gracia J L Rouzaut A Sanmamed M F Le Bon A Melero I Direct effects of type I interferons on cells of the immune system Clin Cancer Res 2011 17 09 2619 2627 10.1158/1078-0432.CCR-10-1114 21372217

[5] Shi W Y Cao C Liu L Interferon α induces the apoptosis of cervical cancer hela cells by activating both the intrinsic mitochondrial pathway and endoplasmic reticulum stress-induced pathway Int J Mol Sci 2016 17 11 1832 10.3390/ijms17111832 27827850 PMC5133833

[6] Pervolaraki K Rastgou Talemi S Albrecht D Bormann F Bamford C Mendoza J L Differential induction of interferon stimulated genes between type I and type III interferons is independent of interferon receptor abundance PLoS Pathog 2018 14 11 e1007420 10.1371/journal.ppat.1007420 30485383 PMC6287881

[7] Ousman S S Wang J Campbell I L Differential regulation of interferon regulatory factor (IRF)-7 and IRF-9 gene expression in the central nervous system during viral infection J Virol 2005 79 12 7514 7527 10.1128/JVI.79.12.7514-7527.2005 15919906 PMC1143633

[8] Levy D E Marié I Prakash A Ringing the interferon alarm: differential regulation of gene expression at the interface between innate and adaptive immunity Curr Opin Immunol 2003 15 01 52 58 10.1016/s0952-7915(02)00011-0 12495733

[9] Yang M Chen H Zhou L Huang X Su F Wang P Identification of SOCS family members with prognostic values in human ovarian cancer Am J Transl Res 2020 12 05 1824 1838 32509179 PMC7269991

[10] Yoshimura A Nishinakamura H Matsumura Y Hanada T Negative regulation of cytokine signaling and immune responses by SOCS proteins Arthritis Res Ther 2005 7 03 100 110 10.1186/ar1741 15899058 PMC1174965

[11] Severa M Remoli M E Giacomini E Ragimbeau J Lande R Uzé G Differential responsiveness to IFN-alpha and IFN-beta of human mature DC through modulation of IFNAR expression J Leukoc Biol 2006 79 06 1286 1294 10.1189/jlb.1205742 16624932

[12] Tirone N R Peghini B C Barcelos A C Murta E F Michelin M A Local expression of interferon-alpha and interferon receptors in cervical intraepithelial neoplasia Cancer Immunol Immunother 2009 58 12 2003 2010 10.1007/s00262-009-0707-6 19381629 PMC11030549

[13] Bhatla N Aoki D Sharma D N Sankaranarayanan R Cancer of the cervix uteri Int J Gynaecol Obstet 2018 143 02 22 36 10.1002/ijgo.12611 30306584

[14] Moraga I Harari D Schreiber G Uzé G Pellegrini S Receptor density is key to the alpha2/beta interferon differential activities Mol Cell Biol 2009 29 17 4778 4787 10.1128/MCB.01808-0 19564411 PMC2725717

[15] Marrero-Rodríguez D Baeza-Xochihua V Taniguchi-Ponciano K Huerta-Padilla V Ponce-Navarrete G Mantilla A Interferon epsilon mRNA expression could represent a potential molecular marker in cervical cancer Int J Clin Exp Pathol 2018 11 04 1979 1988 31938304 PMC6958218

[16] Pestka S Krause C D Walter M R Interferons, interferon-like cytokines, and their receptors Immunol Rev 2004 202 8 32 10.1111/j.0105-2896.2004.00204.x 15546383

[17] Scott M L Woodby B L Ulicny J Raikhy G Orr A W Songock W K Human papillomavirus 16 E5 inhibits interferon signaling and supports episomal viral maintenance J Virol 2020 94 02 e01582 e19 10.1128/JVI.01582-19 31666385 PMC6955282

[18] Inagaki-Ohara K Kondo T Ito M Yoshimura A SOCS, inflammation, and cancer JAK-STAT 2013 2 03 e24053 10.4161/jkst.24053 24069550 PMC3772102

[19] Sarmah N Baruah M N Baruah S Immune modulation in HLA-G expressing head and neck squamous cell carcinoma in relation to human papilloma virus positivity: a study from Northeast India Front Oncol 2019 9 58 10.3389/fonc.2019.00058 30859089 PMC6397850

[20] Marchetti M Monier M N Fradagrada A Mitchell K Baychelier F Eid P Stat-mediated signaling induced by type I and type II interferons (IFNs) is differentially controlled through lipid microdomain association and clathrin-dependent endocytosis of IFN receptors Mol Biol Cell 2006 17 07 2896 2909 10.1091/mbc.e06-01-0076 16624862 PMC1483027

[21] Peghini B C Abdalla D R Barcelos A C Teodoro Ld Murta E F Michelin M A Local cytokine profiles of patients with cervical intraepithelial and invasive neoplasia Hum Immunol 2012 73 09 920 926 10.1016/j.humimm.2012.06.003 22749886

